# Comparison of Serologic Assays for Middle East Respiratory Syndrome Coronavirus

**DOI:** 10.3201/eid2510.190497

**Published:** 2019-10

**Authors:** Ruth Harvey, Giada Mattiuzzo, Mark Hassall, Andrea Sieberg, Marcel A. Müller, Christian Drosten, Peter Rigsby, Christopher J. Oxenford, study participants

**Affiliations:** National Institute for Biological Standards and Control—MHRA, Potters Bar, UK (R. Harvey, G. Mattiuzzo, M. Hassall, P. Rigsby);; Charité-Universitätsmedizin Berlin, Berlin, Germany (A. Sieberg, M.A. Müller, C. Drosten);; German Centre for Infection Research, Berlin (M.A. Müller, C. Drosten);; World Health Organization, Lyon, France (C.J. Oxenford).

**Keywords:** Middle East respiratory syndrome coronavirus, MERS-CoV, antibodies, diagnostics, standard, serology, viruses, Oman, Saudi Arabia, South Korea

## Abstract

Middle East respiratory syndrome coronavirus (MERS-CoV) was detected in humans in 2012. Since then, sporadic outbreaks with primary transmission through dromedary camels to humans and outbreaks in healthcare settings have shown that MERS-CoV continues to pose a threat to human health. Several serologic assays for MERS-CoV have been developed globally. We describe a collaborative study to investigate the comparability of serologic assays for MERS-CoV and assess any benefit associated with the introduction of a standard reference reagent for MERS-CoV serology. Our study findings indicate that, when possible, laboratories should use a testing algorithm including >2 tests to ensure correct diagnosis of MERS-CoV. We also demonstrate that the use of a reference reagent greatly improves the agreement between assays, enabling more consistent and therefore more meaningful comparisons between results.

Since the emergence of Middle East respiratory syndrome coronavirus (MERS-CoV) in 2012 (*1*), more than 2,250 laboratory-confirmed cases have been reported to the World Health Organization (WHO); approximately one third of these cases were fatal. A large proportion of MERS-CoV cases have been the result of human-to-human transmission in healthcare settings (*2*,*3*); outbreaks have occurred in several countries, with the largest outbreaks seen in Saudi Arabia, United Arab Emirates, and South Korea (*4*). Dromedary camels are the putative reservoir hosts for MERS-CoV; they experience no or mild symptoms upon infection (*5*). Primary infection can occur from dromedary camels to humans, and new cases with evidence of camel contact continue to occur sporadically (*6*). MERS-CoV is 1 of the 10 high-threat pathogens on the WHO Research and Development Blueprint (*7*), a document that sets out a roadmap for research and development of diagnostics, preventive and therapeutic products for prevention, and early detection and response to these high-priority pathogens. The WHO roadmap for MERS-CoV lists several priority activities, including improved diagnostics and vaccines for humans and camels as well as basic and translational research (*8*). Serologic assays are critical for the evaluation of the efficacy of new vaccines and patient treatment, as are diagnostic tools to confirm infections and perform serosurveillance. A variety of serologic assays have been developed globally, both commercially and in-house; however, there is no evidence supporting the quality of performance of these assays and their consistency with one another. Participants at the WHO intercountry meeting on MERS-CoV in Cairo, Egypt, June 20–22, 2013, recognized this issue as a public health priority and called for a study to compare currently available serologic assays (*9*). Therefore, we assembled a panel of human serum or plasma and polyclonal antibodies to compare the performance of serologic assays for MERS-CoV. We invited participants to use their testing algorithms to diagnose each sample as if it were a real patient sample. The assays were evaluated for sensitivity and specificity. Pas et al. described in 2015 the impact that a single international standard would have on reducing interlaboratory variability for MERS-CoV diagnostics (albeit in this case for NAT assays) (*10*). To this end, we included 2 samples in the panel as examples of potential WHO International Standard material, and we assessed their effectiveness in harmonization of the data from the participant laboratories.

## Methods

### Study Samples

The National Institute for Biological Standards and Control (NIBSC) Human Material Advisory Committee (project 16/005MP) approved this project. The Ministry of Health, Oman; Ministry of Health, Saudi Arabia; and Korea National Institute of Health, South Korea, donated convalescent serum and plasma samples from PCR-confirmed MERS-CoV–infected patients. All patient donors gave informed consent for the use of their serum or plasma, and samples were anonymized.

We treated all MERS-CoV convalescent-phase serum and plasma with solvent detergent using a validated method (*11*) to ensure there was no residual infectious virus. We stored all study samples at −20°C until dispatched on ice packs to participants. We blind coded the study samples provided to the participants (Table 1).

**Table 1:** Samples used in study of serologic assays for MERS-CoV*

No.	Name	Description	Expected result
1	Korea 5	Single plasma from laboratory-confirmed MERS patient	Positive
2	Tc Bovine NC	Purified IgG from transchromosomal bovine, negative control	Negative
3	WHO/B	Negative control serum, high titer for other CoV	Negative
4	Tc Bovine SAB 300	Purified IgG from transchromosomal bovine, antigen whole virus	Positive
5	Korea 2	Single plasma from laboratory-confirmed MERS patient	Positive
6	WHO/G	Negative control serum, high titer for other CoV	Negative
7	WHO/A	Negative control serum, high titer for other CoV	Negative
8	WHO/D	Negative control serum, high titer for other CoV	Negative
9	Korea 3	Single plasma from laboratory-confirmed MERS patient	Positive
10	Tc Bovine SAB 301	Purified IgG from transchromosomal bovine, antigen spike protein	Positive
11	Korea 1	Single plasma from laboratory-confirmed MERS patient	Positive
12	Korea 4	Single plasma from laboratory-confirmed MERS patient	Positive
13	WHO/F	Negative control serum, high titer for other CoV	Negative
14	Pool C (low)	Pooled serum samples from laboratory-confirmed MERS patients	Positive
15	WHO/C	Negative control serum, high titer for other CoV	Negative
16	Pool A (high)	Pooled serum samples from laboratory-confirmed MERS patients	Positive
17	WHO/E	Negative control serum, high titer for other CoV	Negative
18	Pool B (medium)	Pooled serum samples from laboratory-confirmed MERS patients	Positive

*All samples were submitted as liquid in screw-cap tubes. CoV, coronavirus; MERS, Middle East respiratory syndrome; Tc, transchromosomal; WHO, World Health Organization.

**Figure 1:**
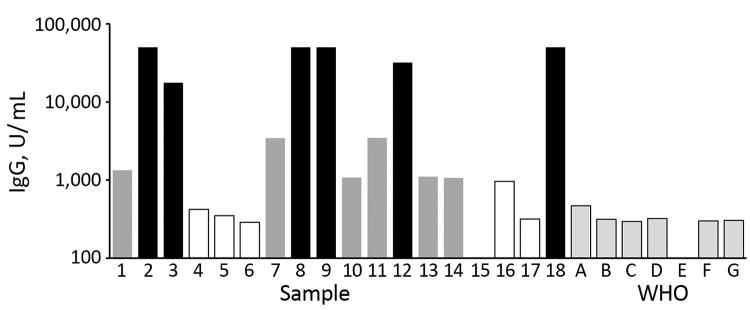
Pooling of serum samples based on their ELISA titers in study of serologic assays for Middle East respiratory syndrome coronavirus. Bar shading indicates the mean ELISA unit value of 2 independent experiments run in duplicate. Black bars represent samples used in pool A (high-positive); dark gray bars indicate samples used in pool B (medium-positive); white bars, and sample 15 with no visible bar, indicate samples used in pool C (low-positive). Pale gray bars with black outline indicate results from a set of negative control samples (WHO A–G). WHO, World Health Organization.

We included 5 plasma samples (samples 1, 5, 9, 11, and 12) as individual patient samples. Other smaller serum donations were pooled in 3 samples (samples 14, 16, and 18) based on their antibody titer, which we determined using the Recombivirus human MERS-CoV nucleoprotein (NP) antibody (IgG) ELISA kit (Alpha Diagnostic International, https://4adi.com). We categorized samples into high-, medium-, or low-positive pools (Figure 1).

We included MERS-CoV–negative serum with antibodies against other human coronavirus HCoV-229E, HCoV-NL63, HCoV-OC43, and HCoV-HKU1 (samples 3, 6, 7, 8, 13, 15, and 17) to test specificity of the assays (Table 2). Co-authors A.S., M.A.M., and C.D. precharacterized and donated these samples. Purified human MERS polyclonal antibodies from transchromosomal (Tc) cattle (*12*) immunized with either recombinant spike protein or whole inactivated virus (samples 2, 4, and 10) were donated by Eddie J. Sullivan (SAB Biotherapeutics, Inc., Sioux Falls, SD, USA). We diluted the material in universal buffer (10 mmol/L Tris-HCl, pH 7.4, 0.5% human serum albumin, 2% trehalose) at a concentration of 1 mg/mL.

**Table 2:** Characterization of MERS-CoV–negative control serum panel included in study of serologic assays for MERS-CoV*

Name	Recombinant spike protein–based indirect immunofluorescence test, reciprocal endpoint titers*					
	HCoV-229E	HCoV-NL63	HCoV-OC43	HCoV-HKU1	SARS-CoV	MERS-CoV
WHO/A	160	1,280	320	640	NR	NR
WHO/B	2,560	1,280	1,280	160	NR	NR
WHO/C	160	320	1,280	320	NR	NR
WHO/D	1,280	2,560	320	160	NR	NR
WHO/E	320	1,280	160	160	NR	NR
WHO/F	80	320	320	160	NR	NR
WHO/G	320	320	1,280	1,280	NR	NR

*All serum samples were tested in a dilution range of 1:20 to 1:5,120. CoV, coronavirus; HCoV, human CoV; MERS, Middle East respiratory syndrome; NR, nonreactive at the cutoff serum dilution of 1:20; SARS, severe acute respiratory syndrome; WHO, World Health Organization.

### Participants

The 10 participating laboratories (listed at the end of this article) were located in Australia, China (2 mainland, 1 Hong Kong), Germany, South Korea, the Netherlands, United States, and the United Kingdom (2 laboratories). Participating organizations included national control laboratories, diagnostic laboratories, and research laboratories.

### Study Protocol

Participants tested the sample panel using their routine assays for MERS-CoV serology. We asked participants to perform 3 independent assays on different days. We provided an Excel spreadsheet (Microsoft, http://www.microsoft.com) for reporting the raw data for each assay and any interpretation of the results, such as positive or negative diagnosis of the samples.

### Statistical Methods

We combined titers and relative potency (relative titer) estimates as unweighted geometric means (GMs) for each sample and laboratory and used these laboratory GMs to calculate overall unweighted GMs for each sample. We expressed variability between laboratories using geometric coefficients of variation (GCV = [10*^s^* − 1]×100%, where *s* is the SD of log_10_ transformed estimates). To mitigate the effect of any potential outliers, we calculated robust estimates of *s* using the R package WRS2 (*13*).

### Coding of Returned Data 

We referred to all participating laboratories by code numbers, which were randomly allocated. If a laboratory returned data using different assay methods, we assayed the results separately for each method and referred to them according to their laboratory number and assay code; for example, 04 ppNT (pseudoparticle neutralization test) and 04 TCID_50_ (50% tissue culture infectious dose).

## Results

A total of 27 datasets were returned (Table 3). Data covered a range of different assay formats: neutralization assays, ELISA, immunofluorescence tests, and 1 microarray. In general, there was good agreement between all the assays tested in this study. In assays with a quantitative measurement, the limit of detection and titer of samples varied greatly, but overall determination of positive or negative agreed between all assays except for 1 (laboratory 04 TCID_50_ MN [microneutralization]), which failed to detect 2 positive samples (samples 9 and 18) that all other tests detected as positive.

**Table 3:** Summary of all data returned in collaborative study of serologic assays for MERS-CoV*

Assay type	Lab	Method	1	2	3	4	5	6	7	8	9	10	11	12	13	14	15	16	17	18
Endpoint																				
ELISA	01	S1 ELISA†	3,200	<100	<100	3,200	6,400	<100	<100	<100	800	1,600	1,600	1,600	<100	<100	<100	5,600	<100	1200
ELISA	03	Primary screening EIA	81,920	<20	<20	51,200	>327,680	<20	<20	<20	5,120	20,480	20,480	20,480	<20	<20	<20	81,920	<20	3200
Neut	01	PRNT	1,280	<20	<20	320	1,280	<20	<20	<20	80	160	160	320	<20	<20	<20	640	<20	80
Neut	03	MERS wt MN	1,280	<10	<10	80	1,280	<10	<10	<10	40	160	80	80	<10	<10	<10	160	<10	40
Neut	04	ppNT	1,280	<10	<10	160	160	<10	<10	<10	10	80	40	40	<10	<10	<10	80	<10	10
Neut	04	TCID_50_ MN	160	<10	<10	80	40	<10	<10	<10	<10	40	20	10	<10	<10	<10	40	<10	<10
Neut	04	PRNT (ED_50_)	>320	<10	<10	160	>320	<10	<10	<10	80	160	80	80	<10	<10	<10	160	<10	20
Neut	04	PRNT (ED_90_)	>320	<10	<10	80	80	<10	<10	<10	20	80	40	20	<10	<10	<10	80	<10	10
Neut	05	PRNT	2,932	<100	<100	1,111	>6,400	<100	<100	<100	444	1,010	3,284	804	<100	<100	<100	1,313	<100	933
Neut	06	PRNT	640	<20	<20	320	640	<20	<20	<20	226	320	320	452	<20	<20	<20	905	<20	113
Neut	08	ppNT	10,240	<10	<10	320	1,280	<10	<10	<10	80	320	160	320	<10	<10	<10	640	<10	80
Neut	10	PRNT (ED_90_)	1,626	<32	<32	256	645	<32	<32	<32	64	256	102	64	<32	<32	<32	645	<32	102
Other	01	S1 microarray	231	<20	<20	1,152	1,251	<20	<20	<20	226	676	681	463	<20	<20	<20	785	<20	90
Qualitative																				
ELISA	01	S1 ELISA‡	BL/P	N	N	P	P	N	N	N	BL/N	P	P	P	N	N	N	P	N	BL
ELISA	02	N titration	P	N	N	N	P	N	N	N	P	N	P	P	N	N	N	P	N	P
ELISA	02	S titration	P	N	N	P	P	N	N	N	P	P	P	P	N	N	N	P	N	P
ELISA	07	ELISA IgG‡	P	N	N	P	P	N	N	N	Equiv	P	P	P	N	N	N	P	N	P
ELISA	09	RBD-based ELISA	P	N	N	P	P	N	N	N	P	P	P	P	N	N	N	P	N	P
ELISA	09	S1 ELISA	P	N	N	P	P	N	N	N	P	P	P	P	N	N	N	P	N	P
ELISA	05	Alpha NP IgG	P	N	N	N	P	N	N	N	P	N	P	P	N	P	N	P	N	P
ELISA	10	ELISA IgG‡	P	N	N	P	P	N	N	N	Weak P	P	P	P	N	N	N	P	N	Weak P
Neut	09	ppNT	P	N	N	P	P	N	N	N	P	P	P	P	N	N	N	P	N	P
Other	03	Secondary screening IFO	P	N	N	P	P	N	N	N	P	P	P	P	N	N	N	P	N	P
Other	06	IIFT‡	P	N	N	P	P	N	N	N	P	P	P	P	N	N	N	P	N	P
Other	06	rIIFT†	P	N	N	P	P	N	N	N	P	P	P	P	N	N	N	P	N	P
Other	07	IF†	P	N	N	P	P	N	N	N	Equiv/P	P	P	P	N	N	N	P	N	P/Equiv
Other	10	IIFT‡	P	N	N	P	P	N	N	N	P	P	P	P	N	N	N	P	N	P

*Sample numbers in gray shading are positive samples, those in white are negative. Green shading indicates correct diagnosis; red shading indicates incorrect diagnosis, yellow indicates borderline or equivocal results. Data shown in the top part of the table are antibody titers as measured in each assay format. BL, borderline; CoV, coronavirus; ED, effective dose; EIA, enzyme immunoassay; Equiv, equivocal; HCoV, human CoV; IF, immunofluorescence; IFO, immunofocus assay; IIFT, indirect immunofluorescence test; MERS, Middle East respiratory syndrome; MN, microneutralization; N, negative; Neut, neutral; P, positive; ppNT, pseudoparticle neutralization test; PRNT, plaque reduction neutralization assay; RBD, receptor-binding domain; rIIFT, recombinant indirect immunofluorescence test; S1, spike protein. †In-house assay. ‡Assay by Euroimmun (https://www.euroimmun.com).

The panel of negative control samples was deemed to be negative in all quantitative assays. There were 3 instances of laboratories reporting a result above cutoff for samples in 1 assay, but these samples were correctly diagnosed as negative overall by their testing algorithms: laboratory 02 detected samples 3 and 7 as above cutoff at 1:80 dilution in 1 assay only; laboratory 02 detected sample 13 as above cutoff at 1:100 and 1:400 dilutions in 1 assay; and laboratory 03 detected sample 13 as above cutoff at 1 dilution tested.

Participants detected pool A, the high-titer MERS-CoV antibody pool (sample 16) in all assays (Table 3). They detected pool B, the medium-titer pool (sample 18), in all but 1 of the quantitative assays, a TCID_50_ MN assay from laboratory 04. In all other quantitative assays, participants detected the high pool at a higher titer than the medium pool. In the qualitative assays, 3 assays gave borderline positive or equivocal results for the medium pool; these assays were a Euroimmun S1 ELISA (https://www.euroimmun.com) in laboratories 01 and 10 and an in-house immunofluorescence assay in laboratory 07. The low-positive pool (pool C, sample 14) was only detected as positive in a single assay in the study, the Alpha Diagnostic International MERS NP ELISA performed in laboratory 05.

In all the qualitative assays, participants scored positive the 2 purified IgG samples from MERS-CoV–immunized transchromosomal bovine samples (samples 4 and 10); however, in 2 of the quantitative assays, N ELISA from laboratory 02 and the Alpha Diagnostic International MERS NP ELISA from laboratory 05, these 2 samples were scored negative. We expected these 2 NP assays to fail to detect sample 10 because this antibody was raised against recombinant MERS spike protein only; however, it was surprising that the assays did not detect sample 4, which was an antibody raised against whole inactivated virus.

**Figure 2:**
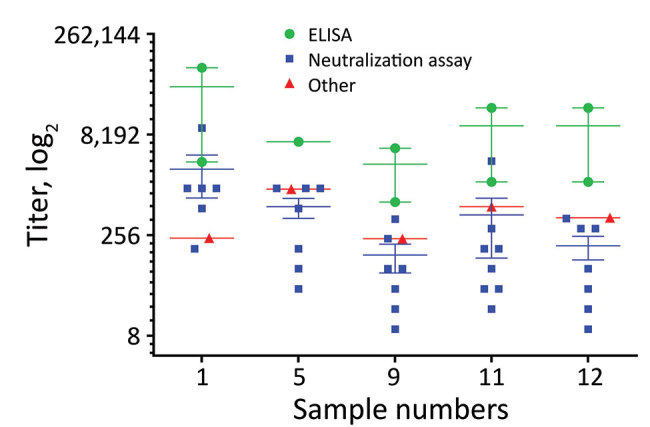
Endpoint titers of individual positive patient plasma samples in study of serologic assays for MERS-CoV. The titers for the 5 individual MERS-CoV–positive patient plasma were determined by ELISA (green circles), neutralization assays (blue squares), and other assays (red triangles). Horizontal lines indicate the mean for each assay type; error bars show SD between assays. MERS-CoV, Middle East respiratory syndrome coronavirus.

For the individual convalescent-phase plasma samples, we saw more variability in the results when compared with the pooled material, but despite this, 3 of the samples (samples 5, 11, and 12) were correctly identified as positive in all tests. Sample 1 was correctly diagnosed as positive in all tests but was identified as borderline positive by laboratory 01 in a Euroimmun ELISA assay. Sample 9 was the most difficult individual patient plasma to detect as positive; it was negative in the TCID_50_ MN assay in laboratory 04; equivocal/positive in the in-house immunofluorescence assay in laboratory 07; and, in the Euroimmun ELISA, borderline/negative in lab 01, equivocal in lab 07, and weak positive in lab 10. Sample 9 was the weakest positive of the individual samples tested in the panel, as we saw from the titers in the quantitative assays that detected it as positive. We compiled the results of quantitative assays for the 5 individual positive plasma samples (Figure 2).

**Figure 3:**
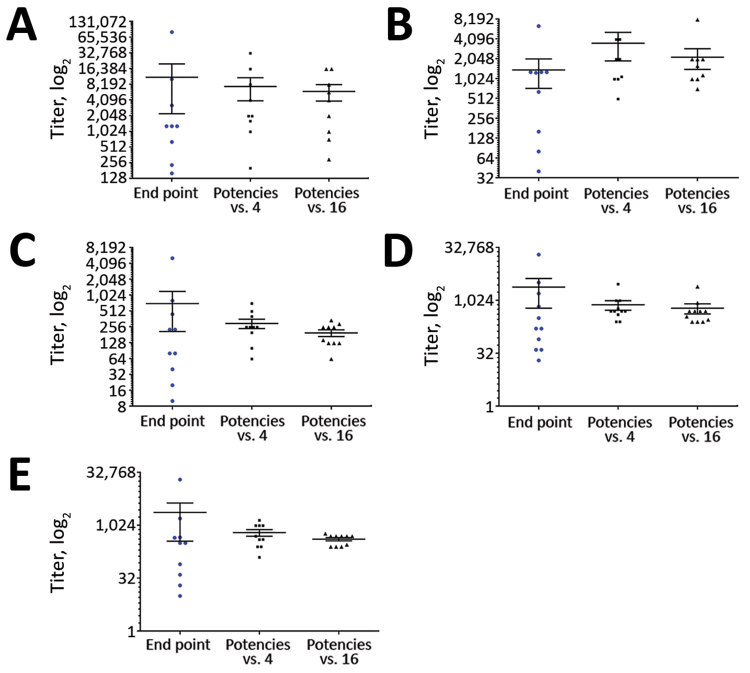
Relative titers of the individual positive patient plasma sample against a reference standard in study of serologic assays for MERS-CoV. Each panel represents a MERS-CoV–positive patient plasma sample: sample 1 (A), sample 5 (B), sample 9 (C), sample 11 (D), sample 12 (E). In each panel, the first data column shows the spread of endpoint titers from all quantitative assays performed; the second and third columns show quantitative results expressed as a potency relative to either sample 4 (Tc Bovine IgG raised against whole virus) or sample 16 (high-positive serum pool A), In each case the sample used as a reference was assigned nominal potency of 1,000 and all other samples were expressed as relative to the reference sample. For each dataset, horizontal line indicates the mean; error bars show SEM. MERS-CoV, Middle East respiratory syndrome coronavirus.

To simplify comparison of the assays, we reported results from the different laboratories as relative to either the transchromosomal bovine IgG sample raised against whole inactivated virus (sample 4) or the high-positive pooled human serum (pool A, sample 16). When we expressed data as a potency relative to either of the 2 chosen potential reference preparations with an assigned arbitrary value of 1,000, we observed improvement in the agreement between tests (Table 4; Figure 3). We saw the greatest reduction in the variation between laboratories (smaller SEM in Figure 3 and smaller percentage geometric coefficient of variation [GCV] in Table 4) when we used pooled human serum (sample 16) as standard. The transchromosomal bovine IgG raised against whole virus (sample 4) showed a substantial improvement in the agreement between laboratories; however, 2 ELISA methods included in this study could not identify this sample as positive.

**Table 4:** GCV percentage (%GCV) for 5 individual serum samples in study of serologic assays for Middle East respiratory syndrome coronavirus*

Sample no.	% GCV		
	Endpoint	Potencies vs. sample 4	Potencies vs. sample 16
1	414	237	342
5	405	173	70
9	555	89	69
11	682	50	73
12	816	138	43

*Values derived from endpoint titers or from potencies expressed as relative to standard samples. GCV, geometric coefficient of variation.

## Discussion

As detection of sporadic cases of MERS-CoV continues, development of new vaccines to combat the disease remains important, as does serosurveillance to understand exposure to the disease and the severity of illness in persons exposed to the virus (*14*). The importance of serologic assays for the diagnosis of MERS cases should also be considered; the WHO guideline for laboratory testing for MERS-CoV, updated in January 2018, specifically includes sample collection from suspected MERS-CoV cases for serology (*15*). The guideline lists 3 situations in which laboratories should conduct serologic testing for MERS-CoV: for defining a sporadic MERS-CoV case if molecular testing, such as nucleic acid amplification methods, is not possible; as part of an investigation of an ongoing outbreak; or serologic surveys, such as retrospective analysis of the extent of an outbreak.

In this collaborative study, we evaluated the performance of assays to detect MERS-CoV antibodies using a panel of serologic samples. All laboratories correctly identified the negative samples in the panel, including those containing antibodies against other coronaviruses, implying good specificity of the assays. Laboratories reported the positive results correctly except for sample 14, which tested negative by all but 1 assay; however, these results demonstrated the importance of a testing algorithm. We observed 14 instances of a sample being incorrectly determined as negative or borderline/equivocal in a single test in a single laboratory (Table 3). However, because each laboratory used a testing algorithm involving >1 method of analysis, all the samples with sporadic spurious results were correctly diagnosed as positive or negative; if they had used a single assay type, we would have found a higher proportion of incorrect results. The results for sample 14, which was a pool of serum samples from patients with confirmed MERS-CoV, highlight a lack of sensitivity in most of the assays in this study; further investigation would be needed to determine why the antibody titers in this pool were below the limit of detection in all but 1 assay targeting N protein. It is important to understand whether there is a specific window of time in which clinical samples for serology should be taken or whether some patients do not mount a detectable antibody response against the spike protein during infection. Such information may lead to further updates of WHO guidelines on laboratory testing for MERS-CoV.

The raw titers reported from the laboratories performing quantitative assays varied greatly, for some samples >1,000-fold, between laboratories (Table 3). The use of a reference material in the assays tightened the values from the laboratories for all the samples, enhanced comparability (Figure 3), and reduced the GCV percentage between all laboratories (Table 4), perhaps unsurprisingly, but the magnitude of reduction in GCV percentage was noteworthy. MERS-CoV is an example of an important emerging pathogen with potential to cause outbreaks; diagnostic tests for emerging pathogens are often developed during outbreaks without proper validation or calibration. 

This study showed the importance of using a standard reagent to allow better comparison of results from different laboratories or interpretation of results from different studies or clinical trials. As we continue to face emerging pathogens, which pose significant risks to human health, it is important to use the experience gained from studies such as this to improve our response to the next threat. Standardizing assays is a key issue when different groups around the world are working to develop and produce novel assays and vaccine products. The need for a standard for MERS-CoV serology was discussed and was widely supported at the WHO–International Vaccine Institute joint symposium for MERS-CoV vaccine development in Seoul, South Korea, June 26–27, 2018. Several potential vaccines are in development, and the immune response elicited, their efficacy, and correlates of protection must be assessed. The use of a reference such as WHO International Standards (*16*) will enable worldwide harmonization of assays and comparability of the results from different preclinical and clinical studies. Assessing the specificity and sensitivity of methods is crucial to improve our understanding of the use and limitations of serologic assays for emerging diseases for which we have little knowledge of disease progression, antibody profiles, and other key information that is available for other infectious diseases.

Study participants who contributed data: L. Caly (Victorian Infectious Diseases Reference Laboratory, Melbourne, Australia); C. Li (National Institutes for Food and Drug Control, Beijing, China); L. Zhao and W. Tan (National Institute for Viral Disease Control and Prevention, Chinese Center for Disease Control and Prevention, Beijing, China); M. Peiris and M. Perera (School of Public Health, The University of Hong Kong, Hong Kong, China); M. Müller and C. Drosten (Institute of Virology, Charité–Universitätsmedizin Berlin, Berlin, Germany); C. Kang and J.-S. Wang (Korea Centers for Disease Control and Prevention, Center for Laboratory Control of Infectious Diseases, Chungcheonhbuk-do, South Korea); B. Haagmans and N.M.A. Okba (Department of Viroscience, Erasmus Medical Center, Rotterdam, the Netherlands); R. Gopal (High-Containment Microbiology, National Infection Service, Public Health England – Colindale, London, UK); S. Myhill and G. Mattiuzzo (National Institute for Biologic Standards and Control, South Mimms, UK); N. Thornburg (Centers for Disease Control and Prevention, Atlanta, Georgia, USA). 
